# Angiography-derived versus wire-based fractional flow reserve for guiding coronary revascularization: a meta-analysis of randomized controlled trials

**DOI:** 10.3389/fcvm.2026.1969354

**Published:** 2026-10-30

**Authors:** Peng-Yu Zhong, Qing-Yi Yang, Liu Lei, Lin He, Wei Ni

**Affiliations:** 1 Department of Cardiology, Beijing Anzhen Nanchong Hospital of Capital Medical University & Nanchong Central Hospital (The Second Clinical College of North Sichuan Medical College), Nanchong, Sichuan, China; 2 The Second Clinical College of North Sichuan Medical College, Nanchong, Sichuan, China

**Keywords:** angiography-derived FFR, coronary artery disease, fractional flow reserve, meta-analysis, percutaneous coronary intervention, QFR

## Abstract

**Background:**

Invasive pressure-wire-based fractional flow reserve (FFR) is the reference standard for physiology-guided coronary revascularization, yet it requires pressure-wire equipment and adenosine-induced hyperemia. Angiography-derived FFR enables functional assessment solely from coronary angiograms, while quantitative pooled evidence from patient-level randomized controlled trials (RCTs) remains limited.

**Methods:**

We searched PubMed, Cochrane Library, Embase and Web of Science Core Collection for eligible RCTs comparing angiography-derived FFR-guided versus wire-based FFR-guided revascularization. The primary endpoint was major adverse cardiovascular events (MACE). Pooled risk ratios or mean differences with 95% confidence intervals were calculated. GRADE assessment and trial sequential analysis (TSA) were performed, and publication bias was evaluated by funnel-plot inspection.

**Results:**

Four RCTs involving 6,542 participants were included. No significant difference in MACE was observed between groups (RR 1.07, 95%CI 0.91-1.27). All-cause mortality, cardiac mortality, recurrent myocardial infarction, unplanned revascularization and stent thrombosis were also comparable. Angiography-derived FFR was associated with higher patients-level PCI rate (RR 1.24, 95%CI 1.15-1.33), lesion-level PCI rate (RR 1.24, 95%CI 1.18-1.31) and shorter procedural time (MD −4.09 min, 95%CI −5.76 to −2.43). TSA showed insufficient sample size for most hard clinical endpoints. GRADE certainty varied across endpoints, procedural outcomes were rated high-certainty, while most clinical outcomes were of moderate certainty and stent thrombosis of low certainty.

**Conclusions:**

Relative to wire-based FFR, angiography-derived FFR showed no statistically significant difference in ischaemic-driven clinical outcomes and procedural duration. However, it may increase the likelihood of undergoing PCI. Further large-scale trials with extended follow-up are needed to clarify the long-term implications of this revascularization pattern.

**Systematic Review Registration:**

https://www.crd.york.ac.uk/PROSPERO/, CRD420261479620.

## Introduction

For patients presenting with intermediate coronary artery stenosis, intracoronary pressure-wire-derived fractional flow reserve (FFR) serves as the gold-standard physiological tool for guiding revascularization decisions (1). Randomized evidence has validated that a pressure-wire-guided physiological strategy can correctly identify functionally significant coronary lesions, avoid superfluous percutaneous coronary revascularization (PCI) procedures, and confer favorable long-term clinical outcomes (1). Percutaneous coronary revascularization represents an important therapeutic modality for improving symptomatic status and clinical outcomes among patients with chronic coronary syndromes (2). Nevertheless, real-world clinical uptake of pressure-wire FFR remains suboptimal. Its practical deployment mandates intracoronary wire placement and frequently adenosine-induced hyperemia, bringing extra procedural complexity, patient discomfort, potential wire-related vascular injury and incremental healthcare-related costs (3, 4).

Angiography-derived FFR represents a wire-free alternative physiological modality. It reconstructs three-dimensional coronary vessel anatomy from standard angiographic projections and computes pressure gradients by dedicated computational algorithms, eliminating requirements for pressure-wire instrumentation or vasodilator-induced hyperemia (5, 6). Multiple diagnostic validation investigations have documented acceptable diagnostic agreement between angiography-derived FFR and conventional pressure-wire FFR, implying its potential to streamline catheter-lab workflows and expand access to physiological lesion assessment (5, 6). Multiple distinct commercial software platforms including QFR, vFFR and FFRangio have been developed. Despite disparities in their computational assumptions, they share the same core technical foundation to compute hemodynamic metrics purely from angiographic images, obviating pressure-wires and drug-induced hyperemia. Therefore, robust patient-oriented randomized outcome evidence is required prior to widespread clinical adoption (7).

While diagnostic performances appear promising across validation cohorts, contemporary randomized trials have yielded conflicting patient-level clinical results comparing angiography-derived FFR-guided revascularization against conventional pressure-wire-based guidance (8–11). The FAVOR III Europe trial demonstrated that QFR-driven strategy failed to satisfy prespecified non-inferiority criteria versus pressure-wire FFR. This trial also reported higher rates of PCI alongside a numerically higher MACE event rate, drawing attention to the performance of this specific algorithm (8). Contrasting findings were observed in two subsequent large-scale international randomized studies: FAST-III and ALL-RISE. Both trials established non-inferior clinical endpoints for angiography-derived FFR at 12-month follow-up and reported potential procedural-related advantages such as shortened procedural or fluoroscopy-related time (9, 10). The smaller-sample PROVISION randomized study also assessed FFRangio-guided decision-making within Japanese stable-CAD populations, although MACE was designated as an exploratory endpoint in this investigation (11). Therefore, these four modern randomized controlled trials constitute the complete available randomized evidence base comparing angiography-derived FFR with pressure-wire-based physiology for guiding coronary revascularization. Still, notable methodological and clinical heterogeneities exist across individual studies (12–14).

Current clinical practice guidelines assign Class I recommendation for pressure-wire-based FFR to guide revascularization decisions for intermediate coronary stenosis (15). According to the 2024 ESC CCS guidelines, QFR has a Class I (Level B) recommendation, other angiography-derived FFR systems (vFFR, FFRangio) are Class IIb (Level C), reserved for situations where pressure-wire assessment cannot be performed (15). Nevertheless, guidelines lack dedicated direct comparative evidence evaluating angiography-derived FFR against conventional wire-based FFR.

Accordingly, we conducted this systematic review and meta-analysis of randomized controlled trials aiming to contrast clinical and procedural outcomes of revascularization guided by angiography-derived FFR versus pressure-wire-based coronary physiology for patients with intermediate coronary stenosis. This study was performed to furnish comprehensive evidence supporting clinical decision-making (16).

## Method

### Protocol and registration

This systematic review and meta-analysis was designed and reported strictly in accordance with the Preferred Reporting Items for Systematic Reviews and Meta-Analyses (PRISMA 2020) statement (17). The study protocol was registered on the PROSPERO international prospective register of systematic reviews (CRD 420261479620).

### Search strategy

Four electronic databases including PubMed, Embase, Web of Science Core Collection and Cochrane Library were systematically searched from database inception to October 1, 2026. Search terms combined synonyms of angiography-derived fractional flow reserve (QFR, cFFR, μQFR), invasive wire-based fractional flow reserve, coronary stenosis, percutaneous coronary intervention, and randomized trials. The complete detailed search formula for each database is presented in Supplementary Table S1. Reference lists of previously published relevant meta-analyses and all included randomized controlled trials (RCTs) were manually screened to capture potentially eligible publications. Conference abstracts without publicly available full-text trial data were excluded. No language or publication-date restrictions were applied.

### Inclusion and exclusion criteria

This meta-analysis adopted the PICO eligibility criteria as follows. The study population comprised adult patients (≥18 years) with coronary artery stenosis who were scheduled for functional assessment to guide revascularization. Trials enrolling patients with contraindications to coronary angiography, complex congenital coronary disease or previous coronary artery bypass grafting were excluded. The intervention strategy was revascularization decision-making guided by angiography-derived FFR (QFR/cFFR/μQFR). The comparator group received functional assessment guided by invasive pressure-wire-based FFR for clinical decision-making (FFR/iFR). The primary efficacy endpoint was major adverse cardiovascular events (MACE), defined according to each original RCT protocol. Secondary endpoints comprised lesion-level PCI performance, total procedural time, all-cause mortality, cardiac mortality, recurrent myocardial infarction, unplanned revascularization and stent thrombosis. Eligible study designs were prospective patient-level randomized controlled trials. Non-patient-level randomized trials, observational registries, retrospective cohorts, single-arm studies and non-randomized comparative investigations were excluded to avoid selection bias.

### Data extraction and outcome assessments

Two independent reviewers screened all titles and abstracts, followed by full-text evaluation of potentially eligible citations. Any disagreements regarding study eligibility were resolved via joint discussion with a third senior cardiovascular reviewer (18). Standardized pre-designed data-extraction spreadsheets were used to extract information from each included RCT: (1) basic trial characteristics: first author, publication year, trial acronym, total sample size, number of participants in each study arm, functional assessment protocol, follow-up duration; (2) baseline demographic and clinical features: age, proportion of male patients, prevalence of hypertension, diabetes mellitus, dyslipidaemia and prior cardiovascular disease.

The primary endpoint MACE was extracted as defined by each original trial protocol. Prespecified secondary outcomes included procedural endpoints (Patient-level PCI rate, lesion-level PCI rate, total procedural time) and clinical endpoints (all-cause mortality, cardiac mortality, recurrent myocardial infarction, unplanned revascularization, stent thrombosis).

### Risk of bias and certainty of evidence assessment

Two independent reviewers evaluated the methodological quality of all included RCTs using the Cochrane Risk-of-Bias 2 (RoB 2) tool (19). Each domain was rated as low risk, some concerns, or high risk according to standardized evaluation criteria. Any inconsistencies between evaluators were discussed and settled by a senior supervising reviewer.

Two other investigators independently graded the certainty of evidence for each pooled outcome using the GRADE (Grading of Recommendations Assessment, Development and Evaluation) system. Evidence certainty was stratified into four tiers: high, moderate, low, and very low. We defined a clinically meaningful relative risk threshold of RR = 0.75 for important benefit and RR = 1.25 for important harm for binary endpoints, following empirical conventions from Cochrane systematic reviews in cardiovascular research. Imprecision was judged as serious when the 95% confidence interval simultaneously crossed the null effect (RR = 1.0) and any of these clinically important thresholds; imprecision was judged as very serious if the confidence interval spanned both clinically meaningful benefit and clinically meaningful harm. Disagreements during GRADE rating were resolved through consultation with a third experienced reviewer (20).

### Statistical analysis

Review Manager 5.4.1 software was applied to pool effect sizes. The Mantel-Haenszel model was adopted for dichotomous outcomes and the inverse-variance model for continuous outcomes. A fixed-effect model was used for endpoints with low inter-study heterogeneity, while a random-effects model was applied for outcomes with moderate-to-high heterogeneity to reduce the influence of between-study variation. Results for binary outcomes were reported as pooled risk ratios (RR) with corresponding 95% confidence intervals (CIs). Continuous outcomes were presented as mean differences (MD) and 95% CIs. Statistical heterogeneity was quantified using the I^2^ statistic. Heterogeneity thresholds were defined as low heterogeneity (I^2^＜25%), moderate heterogeneity (25% ≤ I^2^ ≤ 75%), and substantial heterogeneity (I^2^＞75%) (21).

A sensitivity analysis was restricted to the uniform 12-month follow-up by excluding the 24-month data from the FAVOR III Europe trial, to assess the influence of differing follow-up durations. Given only four eligible RCTs were included, quantitative publication-bias tests such as Egger's or Begg's test were not statistically feasible, we only performed visual inspection of funnel plots.

Trial sequential analysis (TSA) was additionally performed using TSA 0.9.5 software for the primary endpoint MACE and all secondary endpoints to mitigate type-I error risk caused by repeated cumulative meta-analytic comparisons. The a-priori parameters for required information size (RIS) calculation were derived from the FAST-III trial, with a type-I error *α* set at 0.05 and statistical power (1−*β*) set at 0.80. The cumulative Z-score curve was plotted against TSA monitoring boundaries. Crossing the monitoring boundary before attaining RIS suggests that the observed association is statistically robust under currently available data, but does not yield definitive clinical evidence. If the cumulative Z-curve stayed within the inner invalid boundaries and failed to reach the RIS, the neutral pooled effect was considered credible, indicating that current accumulated sample size remained insufficient and further large-scale RCTs were required for definitive verification.

## Results

### Study selection

The electronic database search retrieved 607 records, including 136 from PubMed, 64 from Cochrane Library, 225 from Embase, and 182 from Web of Science Core Collection. After removing 170 duplicate records, 437 unique citations remained for title-and-abstract screening. Of these, 421 records were excluded at the title-and-abstract stage, comprising 10 duplicate reports, 399 non-interventional clinical trials, and 12 other irrelevant records. Sixteen articles proceeded to full-text eligibility assessment. Finally, four randomized controlled trials fulfilled the pre-specified eligibility criteria and were included in the quantitative synthesis (9–11, 22). The PRISMA flow diagram of study selection is presented in Figure 1.

**Figure 1:**
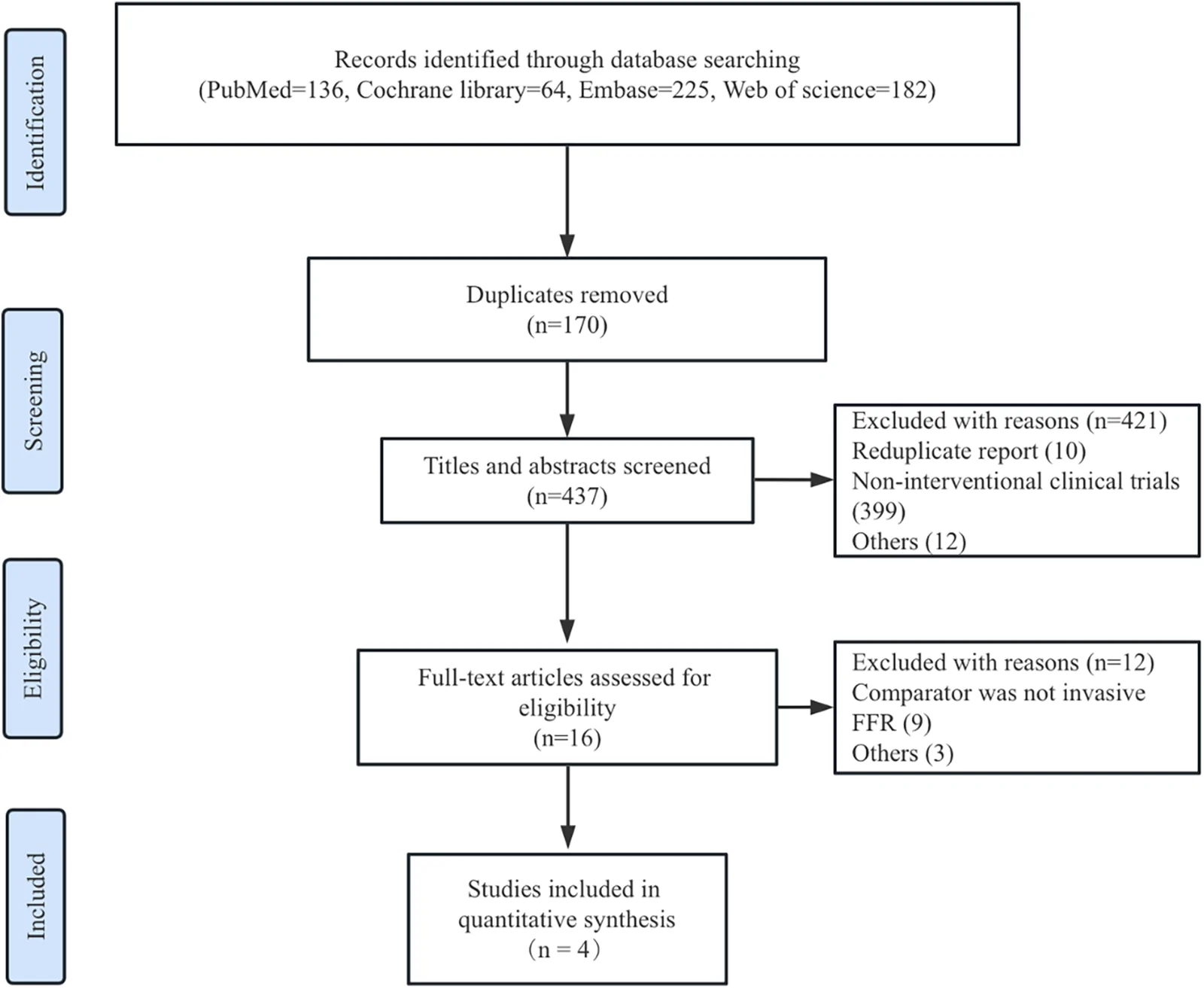
PRISMA flow diagram illustrating the process of study identification, screening, eligibility assessment, and final inclusion in the quantitative synthesis.

### Characteristics of included studies

Four multicentre open-label non-inferiority RCTs involving 6542 participants were included (Table 1). FAVOR III-Europe adopted QFR with 2-year follow-up, FAST-III (vFFR), ALL-RISE (FFRangio) and PROVISION (FFRangio) reported 12-month outcomes. Control-group physiological strategies differed: ALL-RISE allowed either FFR or iFR, while others used adenosine-induced pressure-wire FFR exclusively. Non-inferiority margins and MACE definitions were broadly comparable yet not identical.

**Table 1:** General characteristics of included randomised controlled trials.

Trials	FAVOR III Europe 2026, EuroIntervention	FAST-III 2026, NEJM	ALL-RISE 2026, NEJM	PROVISIONT 2026, JACC-Cardiovasc Interv
Study design	Multicenter, open-label, non-inferiority RCT	Multicenter, open-label, non-inferiority RCT	International multicenter, open-label, non-inferiority RCT	Japanese multicenter, open-label, non-inferiority RCT
Total randomized participants	2000 I = 1008; C = 992	2211 I = 1116; C = 1095	1930 I = 965; C = 965	401 I = 203; C = 198
Study population	CCS, NSTEMI, or staged evaluation of non-culprit lesions post-STEMI; visually estimated 40-90% intermediate stenosis, reference vessel diameter ≥2.5 mm	CCS, unstable angina, NSTE-ACS; 30-80% intermediate stenosis, TIMI-3 flow, reference vessel diameter ≥2.5 mm	Patients ≥18 years old; 50-90% angiographic stenosis requiring physiological assessment; CCS/NSTE-ACS	Patients ≥18 years old, predominantly stable CAD, few UA/NSTEMI; 30-90% stenosis, reference vessel diameter ≥2 mm
Angiography-derived FFR	QFR version 2.0 (Medis)	vFFR (Pie Medical)	FFRangio (CathWorks)	FFRangio (CathWorks)
Control-arm physiological modality	Adenosine-induced pressure-wire FFR only	Adenosine-induced pressure-wire FFR only	FFR or iFR (operator-selected; 44.5% received iFR)	Adenosine-induced pressure-wire FFR only
Decision threshold (I/C)	Revascularization if QFR/FFR ≤0.8; defer if >0.8	Revascularization if vFFR/FFR ≤0.8; defer if >0.8	FFRangio ≤0.8; FFR ≤0.8/iFR ≤0.89	FFRangio ≤0.8; FFR ≤0.8
Key exclusion criteria	STEMI within 24 h, cardiogenic shock, severe renal dysfunction, atrial fibrillation, bypass grafts, CTO, ostial left main stenosis >50%	STEMI within 72 h, cardiogenic shock, left-main disease, adenosine contraindication, bypass grafts	Active STEMI, planned CABG, prior CABG to target vessel, severe valvular disease, severe left ventricular dysfunction	MI within 72 h, severe asthma, eGFR < 45 mL/min/1.73 m^2^, atrial fibrillation, severe vessel tortuosity, myocardial bridging
Non-inferiority margin	3.5 percentage-points for 1-year MACE	3.0 percentage-points for 1-year composite endpoint	3.5 percentage-points for 1-year composite endpoint	14% absolute difference for revascularization rate; MACE as exploratory endpoint
MACE definition	All-cause death + myocardial infarction + unplanned revascularization	All-cause death + any myocardial infarction + any revascularization	All-cause death + myocardial infarction + unplanned clinically-driven revascularization	Cardiac death + myocardial infarction + ischaemia-driven target-vessel revascularization
Follow-up	2-year	12-month	12-month	12-month

C, control group; FFR, fractional flow reserve; I, intervention group; iFR, instantaneous wave-free ratio; QFR, quantitative flow ratio; vFFR, vessel-derived fractional flow reserve. Non-inferiority margins were predefined in each original trial protocol. The full-analysis set was applied for FAST-III. ALL-RISE control arm allowed either pressure-wire FFR or iFR at operator's discretion.

Baseline characteristics are summarized in Table 2. Participants were elderly with substantial cardiovascular risk burdens. Mean age ranged 67.0-71.8 years. Diabetes prevalence was highest in ALL-RISE. ACS and multivessel disease proportions varied notably across trials, with PROVISION enrolling fewer complex patients. Within each trial, baseline clinical and angiographic variables were well-balanced between intervention and control arms.

**Table 2:** Baseline clinical and angiographic patient-level characteristics of included randomised controlled trials.

Characteristic	FAVOR III EuropeAndersen 2026, EuroIntervention	FAST-IIIDaemen 2026, N Engl J Med	ALL-RISEFearon 2026, N Engl J Med	PROVISIONTanigaki 2026, JACC Cardiovasc Interv
Mean age, years (I/C)	67.2/67.0	67.6/67.1	68.4/68.4	71.4/71.8
Female sex, *n* (%) (I/C)	235 (23.3)/227 (22.9)	266 (23.8)/272 (24.8)	236 (24.5)/246 (25.5)	59 (29.1)/56 (28.3)
Diabetes mellitus, *n* (%) (I/C)	256 (25.4)/245 (24.7)	308 (27.6)/281 (25.7)	381 (39.5)/349 (36.3)	72 (35.6)/63 (31.8)
Hypertension, *n* (%) (I/C)	727 (72.1)/712 (71.8)	808 (72.4)/794 (72.5)	757 (78.5)/769 (79.9)	151 (74.4)/145 (73.2)
Dyslipidaemia, *n* (%) (I/C)	746 (74.0)/729 (73.5)	793 (71.1)/784 (71.6)	759 (78.7)/764 (79.4)	148 (72.9)/158 (79.8)
Current smoker, *n* (%) (I/C)	214 (21.2)/207 (20.9)	221 (19.8)/195 (17.8)	150 (15.6)/124 (12.9)	38 (18.7)/26 (13.1)
Prior myocardial infarction, *n* (%) (I/C)	224 (22.2)/211 (21.3)	264 (23.7)/251 (22.9)	166 (17.2)/158 (16.4)	25 (12.3)/29 (14.6)
Prior PCI, *n* (%) (I/C)	404 (40.1)/387 (39.0)	391 (35.0)/380 (34.7)	375 (38.9)/375 (39.0)	59 (29.1)/69 (34.8)
ACS clinical presentation, *n* (%) (I/C)	345 (34.2)/334 (33.7)	205 (18.4)/208 (19.0)	96 (9.9)/85 (8.8)	10 (4.9)/7 (3.5)
Multivessel coronary artery disease, *n* (%) (I/C)	453 (44.9)/437 (44.1)	SYNTAX score >11: 42.0%/41.4%	Not reported	39 (19.2)/42 (21.2)
Left-ventricular ejection fraction, mean % (I/C)	56.1/55.8	54.1/54.6	Not stratified by group	62.2/63.2
Proportion of LAD target lesions, %	49 (all lesions)	49.3 (all lesions)	49.3 (all lesions)	58.6 (I)/66.1 (C)

ACS, acute coronary syndrome; C, control group; I, intervention group; LAD, left anterior descending artery; PCI, percutaneous coronary intervention. Data are presented as mean for continuous variables and *n* (%) for categorical variables. Multivessel disease for FAST-III was reported as proportion of patients with SYNTAX score >11. Left-ventricular ejection fraction was not stratified by study arm in the ALL-RISE trial. Proportion of LAD target lesions was calculated among all physiologically-assessed study lesions.

### Primary outcome

Across four RCTs, 257 out of 3,292 patients in the angiography-derived FFR group experienced MACE, compared with 236 events among 3,250 patients in the pressure-wire FFR group (RR = 1.07, 95%CI: 0.91–1.27, *P* = 0.41). No statistical heterogeneity was detected across trials (P_heterogeneity_=0.39, I^2^ = 0%) (Figure 2). There was no significant difference in the risk of MACE between angiography-derived FFR-guided revascularization and conventional pressure-wire FFR-guided strategy.

**Figure 2:**
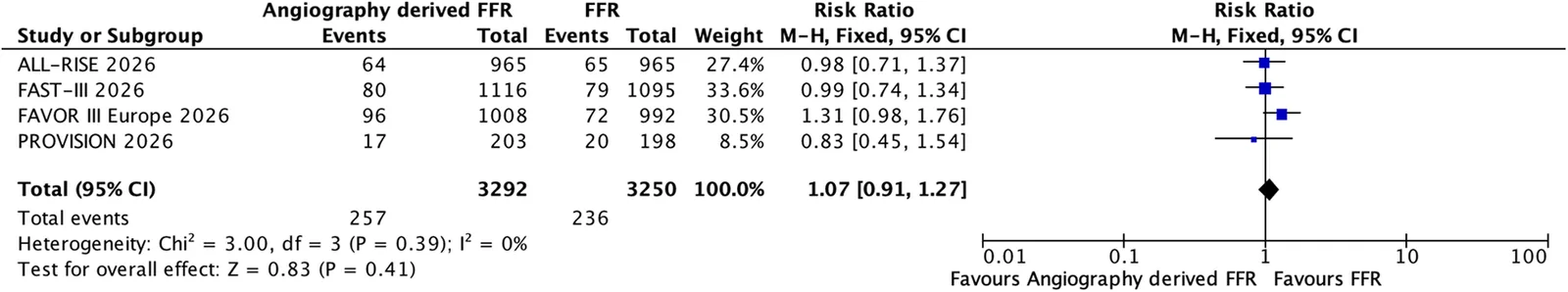
Forest plot comparing angiography-derived FFR-guided revascularization versus pressure-wire FFR-guided strategy for the primary endpoint of major adverse cardiovascular events (MACE). M-H, Mantel-Haenszel; CI, confidence interval.

### Secondary outcomes

For all-cause mortality, 72 out of 3,292 patients in the angiography-derived FFR group experienced death, compared with 68 events among 3,250 patients in the pressure-wire FFR group (RR = 1.05, 95%CI: 0.75–1.45, *P* = 0.79). No statistical heterogeneity was detected across trials (P_heterogeneity_=0.67, I^2^ = 0%) (Figure 3A). For cardiac mortality, 32 events occurred in 3,292 patients receiving angiography-derived FFR-guided therapy versus 36 events among 3,250 patients in the control group (RR = 0.88, 95%CI: 0.55–1.41, *P* = 0.59). Heterogeneity was absent (P_heterogeneity_=0.69, I^2^ = 0%) (Figure 3B). For recurrent myocardial infarction, 103 out of 3,292 patients in the angiography-derived FFR group had recurrent myocardial infarction, compared with 98 events among 3,250 patients in the pressure-wire FFR group (RR = 1.04, 95%CI: 0.79–1.36, *P* = 0.80). Low heterogeneity was observed (P_heterogeneity_=0.34, I^2^ = 11%) (Figure 3C). For unplanned revascularization, 146 events were reported in 3,292 patients in the angiography-derived FFR group versus 142 events among 3,250 patients in the control group (RR = 1.01, 95%CI: 0.81–1.27, *P* = 0.90). No heterogeneity was identified (P_heterogeneity_=0.76, I^2^ = 0%) (Figure 3D). For stent thrombosis, 11 out of 3,089 patients in the angiography-derived FFR group developed stent thrombosis, compared with 12 events among 3,052 patients in the pressure-wire FFR group (RR = 0.91, 95%CI: 0.40–2.05, *P* = 0.81). There was no detectable heterogeneity (P_heterogeneity_=0.57, I^2^ = 0%) (Figure 3E). No significant between-group differences were observed for any of these secondary endpoints.

**Figure 3:**
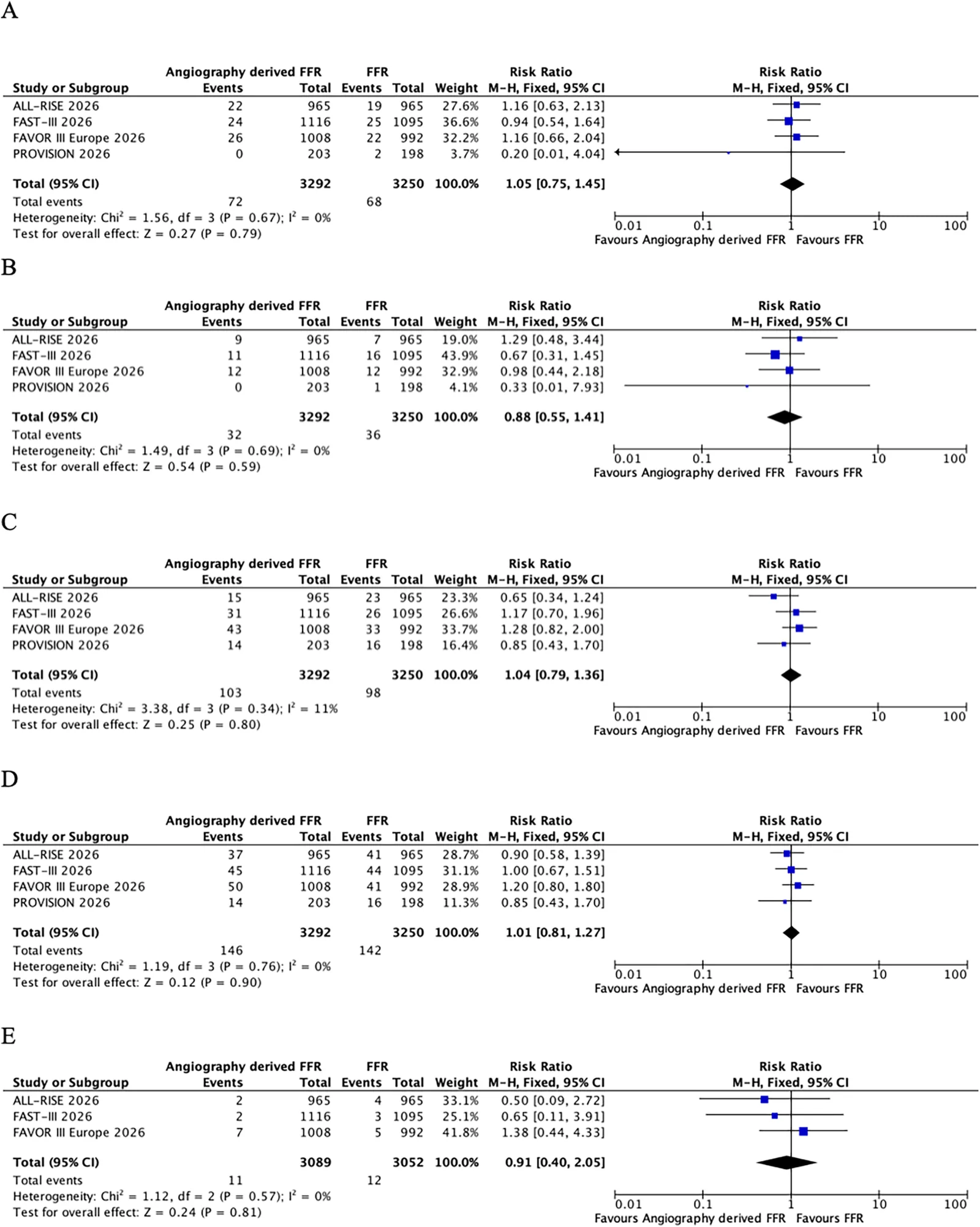
Forest plots for secondary clinical outcomes comparing angiography-derived FFR-guided revascularization versus pressure-wire FFR-guided strategy. **(A)** All-cause mortality. **(B)** Cardiac mortality. **(C)** Recurrent myocardial infarction. **(D)** Unplanned revascularization. **(E)** Stent thrombosis. M-H, Mantel-Haenszel; CI, confidence interval.

In the patient-level analysis of index-procedure PCI (3 trials, *n* = 4,542), angiography-derived FFR was associated with a significantly higher rate of undergoing PCI compared with wire-based FFR (RR 1.24, 95% CI 1.15-1.33, *P* < 0.00001, I^2^ = 0%) (Figure 4A). Across four RCTs, 1,842 out of 4,222 lesions in the angiography-derived FFR group underwent PCI, compared with 1,456 lesions among 4,144 lesions in the pressure-wire FFR group (RR = 1.24, 95%CI: 1.18–1.31, *P* < 0.00001). Low statistical heterogeneity was detected across trials (P_heterogeneity_=0.29, I^2^ = 20%) (Figure 4B). Lesion-level PCI rate was significantly higher in the angiography-derived FFR-guided arm. For total procedural time, pooled data from two RCTs demonstrated that angiography-derived FFR guidance was associated with shorter procedural time (MD = −4.09, 95%CI: −5.76 to −2.43, *P* < 0.00001), with low-to-moderate heterogeneity (P_heterogeneity_=0.22, I^2^ = 34%) (Figure 4C).

**Figure 4:**
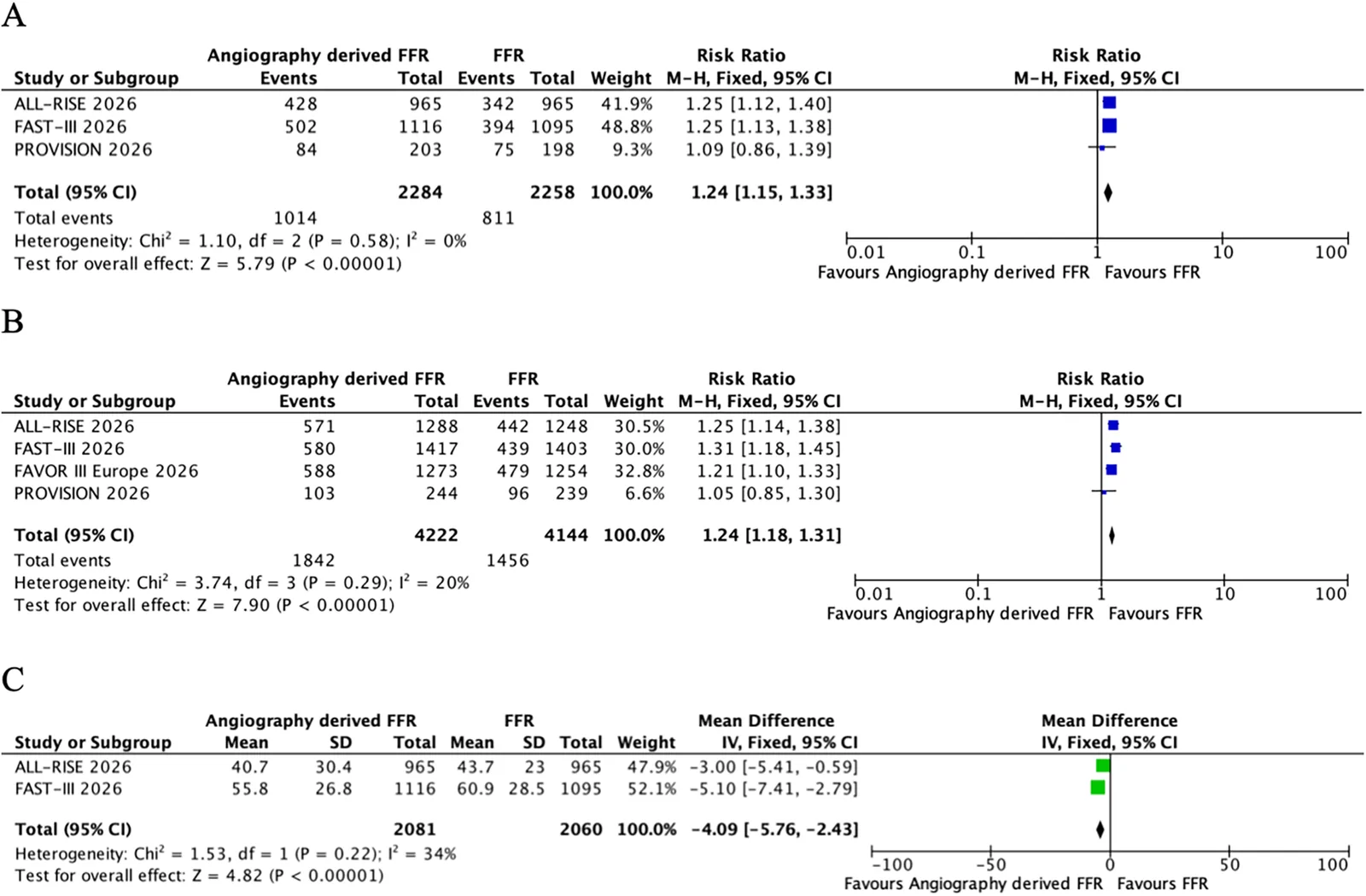
Forest plots for procedural outcomes comparing angiography-derived FFR-guided revascularization versus pressure-wire FFR-guided strategy. **(A)** Patient-level PCI performed **(B)** Lesion-level PCI performed. **(C)** Total procedural time. M-H, Mantel-Haenszel; IV, inverse variance; CI, confidence interval.

### Sensitivity analysis

Sensitivity analysis restricted to uniform 12-month follow-up was performed by excluding the FAVOR III Europe trial (Supplementary Figure S1). After exclusion of FAVOR III Europe, the pooled RR for MACE (RR = 0.97, 95%CI 0.80–1.20), consistent with the primary analysis. Similarly, no statistically significant differences were observed in all-cause mortality (RR = 0.99, 95%CI 0.66–1.48), cardiac mortality (RR = 0.83, 95%CI 0.46–1.48), recurrent myocardial infarction (RR = 0.91, 95%CI 0.64–1.28), unplanned revascularization (RR = 0.94, 95%CI 0.71–1.23) and stent thrombosis (RR 0.57, 95%CI 0.17–1.91). Lesion-level PCI rates remained significantly higher in the angiography-derived FFR group (RR 1.26, 95%CI 1.18–1.34). Collectively, this sensitivity analysis demonstrated that the pooled estimates were robust after restricting analyses to trials with consistent 12-month follow-up.

### Trial sequential analysis

Trial sequential analysis was performed for the primary endpoint MACE and the procedural endpoint patient-level PCI performed (Figure 5). For MACE (Figure 5A), the cumulative Z-curve did not cross the monitoring boundary for benefit or harm, and the required information size was 75,334 participants, indicating that the current sample size was insufficient to draw a definitive conclusion regarding MACE. For patient-level PCI performed (Figure 5B), the cumulative Z-curve crossed the conventional boundary and reached the required information size of 2,406 participants, showing statistical robustness for the observed procedural difference under current data, rather than definitive clinical evidence. Trial sequential analysis was further performed for all secondary endpoints (Supplementary Figure S2). The cumulative Z-curves for all-cause mortality, cardiac mortality, recurrent myocardial infarction, unplanned revascularization and stent thrombosis did not cross any monitoring boundary, and the required information size of 75,334 participants was not reached, suggesting insufficient evidence for definitive conclusions on these secondary outcomes.

**Figure 5:**
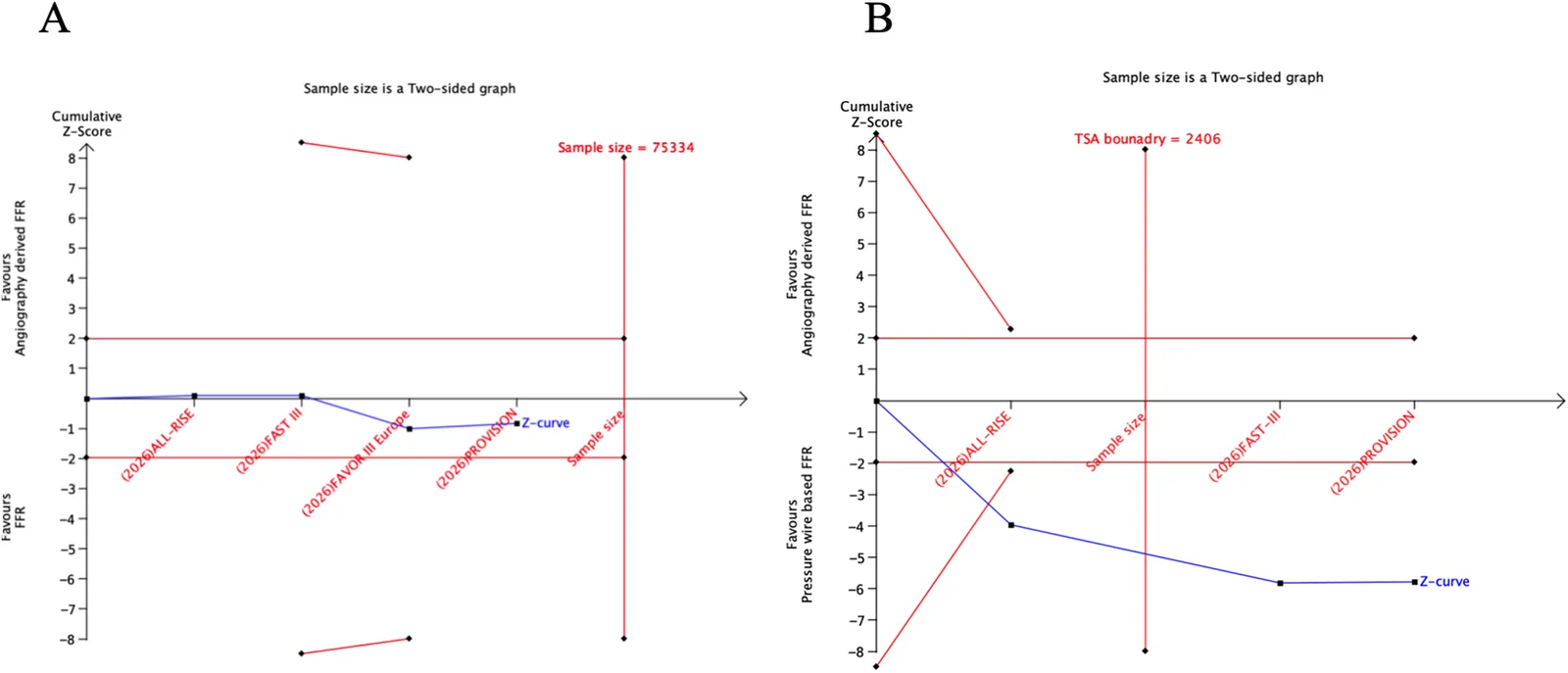
Trial sequential analysis plots. **(A)** Trial sequential analysis for MACE. The cumulative Z-curve failed to cross the benefit-harm monitoring boundary, with a required information size of 75,334 participants. **(B)** Trial sequential analysis for Patient-level PCI performed. The cumulative Z-curve crossed the benefit monitoring boundary prior to achieving the required information size of 2,406 participants. Z-curve, cumulative Z-score curve.

### Assessment of quality and publication bias

RoB-2 risk-of-bias assessment for the primary outcome demonstrated low risk of bias within domains of randomization, missing outcome data, outcome measurement and selective reporting for all four trials. Some concerns were consistently identified for the domain of deviations from intended interventions across all studies, because operators were unblinded to treatment allocation, which may influence revascularization decision-making. Overall risk-of-bias was judged as some concerns for all included randomized trials (Supplementary Table S2). The GRADE framework was applied to assess the certainty of evidence for each pooled outcome. Certainty varied across endpoints: most clinical outcomes were rated as moderate, stent thrombosis as low, whereas patient-level PCI and total procedural time were rated as high certainty. Detailed GRADE evidence-certainty ratings are presented in Supplementary Table S3. Funnel plots were generated to assess publication bias for all pooled outcomes (Supplementary Figure S3). Visual inspection of funnel plots did not show obvious asymmetry. Nevertheless, given the small number of included trials, funnel-plot findings cannot be regarded as evidence for the absence of publication bias.

## Discussion

This meta-analysis synthesizes aggregate outcome data from four patient-level randomized controlled trials, to compare angiography-derived FFR-guided revascularization decisions against conventional pressure-wire-based FFR (9–11, 22). When data were pooled quantitatively, we observed similar risks of the primary endpoint MACE between the two physiological assessment strategies. Secondary clinical endpoints including all-cause mortality, cardiac death, recurrent myocardial infarction, unplanned revascularization and stent thrombosis also showed no measurable inter-group difference. Nevertheless, patients managed by angiography-derived FFR were more likely to receive lesion-level and patient-level PCI, while overall procedural duration was markedly shortened in this cohort. Findings from trial sequential analysis indicated that accumulated sample size had not reached the required information threshold for most hard clinical endpoints, implying firm conclusions for ischemic clinical events still await further evidence accumulation.

In modern interventional cardiology, pressure-wire-based FFR remains the gold-standard invasive physiological tool for identifying ischemia-related coronary stenosis and guiding revascularization choices (23). Despite its well-validated prognostic performance, this conventional technique carries practical downsides. It requires a pressure-sensing guidewire and adenosine-induced hyperemia, increasing costs and prolonging catheter-lab procedures (24). Moreover, adenosine infusion may cause chest pain, dyspnea or transient hypotension in some patients, posing challenges for catheter-lab operators (24). Angiography-derived FFR calculates functional indices solely from routine coronary angiograms without pressure-wire hardware or pharmacological hyperemia, and thus promises wider adoption of physiology-guided revascularization (9–11, 22). Current clinical guideline recommendations for angiography-derived functional assessment are nuanced. The 2024 ESC chronic coronary syndrome guidelines give Class I (Level A) recommendation to pressure-wire-based FFR and iFR for guiding revascularization for intermediate coronary stenoses, whereas among angiography-derived tools, QFR obtains Class I (Level B) and other angiography-derived FFR software receive Class IIb (Level C) recommendations as alternative functional-assessment modalities (15).

Even so, several unresolved clinical questions restrict widespread routine adoption of angiography-derived FFR. First, individual RCTs had separately reported non-inferior clinical outcomes, yet quantitative pooled evidence synthesizing all available randomized data had not previously been generated (9–11, 22). Second, all four included trials consistently demonstrated higher rates of lesion-level PCI within the angiography-derived FFR arm (9–11, 22). This consistent signal raises an important clinical dilemma: whether higher revascularization rates represent correction of undertreated ischemic lesions under wire-FFR assessment or alternatively reflect a tendency toward unnecessary intervention. Notably, the pooled analysis in our study did not detect excess ischemic adverse events despite more frequent PCI procedures, an observation consistent across each source trial. Variations in algorithm-specific ischemia cut-off thresholds among different angiography-derived FFR platforms may partially explain this discrepancy in revascularization frequency (10, 22). Third, although operators gained procedural efficiency with shorter lab time, this procedural benefit failed to translate into measurable improvements in patient-centered hard endpoints in either our pooled analysis or the original randomized investigations (9, 11).

Our pooled findings carry practical implications for daily catheter-lab practice. Angiography-derived FFR can serve as an acceptable alternative physiological assessment strategy under specific clinical scenarios, such as centers with limited access to pressure-wire equipment, or patients who cannot tolerate adenosine-provoked hyperemia (22, 25). Clinicians ought to remain mindful of the tendency toward more frequent lesion-level PCI linked to this angiography-based modality. Even though short-term ischemic event rates were not elevated within existing trial datasets, increased PCI volume may expose patients to additional procedural hazards, higher radiation burden and greater healthcare expenditure; the long-term clinical consequences of this altered revascularization pattern are still incompletely understood. Current guideline recommendations regarding angiography-derived FFR are largely built upon evidence from large-scale individual RCTs. Our meta-analysis provides pooled quantitative evidence showing no statistically significant difference in MACE between the two strategies. Importantly, absence of a statistically-significant difference in MACE cannot constitute formal proof of non-inferiority, given the absence of a pre-specified non-inferiority margin in this meta-analysis.

Looking ahead, larger-scale randomized trials with prolonged follow-up periods are urgently needed. Future research should specifically clarify whether increased PCI utilization observed with angiography-derived FFR reflects appropriate management of ischemic lesions or unwarranted revascularization. Real-world registries will also help characterize the performance of angiography-derived FFR in unselected patient populations, including those presenting with complex multivessel coronary disease (26, 27). Until further high-quality evidence accumulates, clinicians should balance the procedural advantages of angiography-derived FFR against its tendency toward higher revascularization rates when applying this tool in clinical practice.

### Limitations

Several important limitations of the present meta-analysis should be considered when interpreting the findings. First, only four patient-level randomized controlled trials were eligible for quantitative synthesis. Trial sequential analysis indicated that the accumulated sample size for most hard clinical endpoints did not reach the pre-specified required information size. Accordingly, neutral pooled results for mortality, myocardial infarction and stent thrombosis cannot be regarded as definitive. Second, heterogeneity existed across included trials with respect to software versions of angiography-derived FFR algorithms and angiographic image-acquisition protocols, which may contribute to variation in diagnostic performance and revascularization decision-making. Third, blinding of interventional operators was not feasible in these trials, which might introduce ascertainment bias. Despite revascularization decisions relying on quantitative functional values, operators may have lower confidence in Angio-FFR than the reference-standard pressure-wire FFR for borderline lesions, potentially lowering the threshold for stent implantation and partly explaining the higher PCI rate in the Angio-FFR group. Fourth, follow-up durations varied among studies, and robust outcome data beyond 12-month follow-up remain limited. Fifth, our analysis relied exclusively on published aggregate trial data rather than individual-patient data; therefore, we could not perform adjusted subgroup analyses for baseline clinical and angiographic covariates. Sixth, there was heterogeneity in the functional assessment tools adopted across trials: the control arm of the ALL-RISE trial enrolled some patients assessed by iFR in addition to conventional wire-based FFR, while the intervention arms employed multiple angiography-derived functional indices, including QFR, vFFR, and μQFR. Although all these indices are modalities for coronary physiological evaluation, they differ in computational algorithms, diagnostic cut-off values, and adenosine dependence. Finally, the DECISION-QFR trial explored decision-making patterns of QFR versus wire-FFR at Heart-Team level, but randomization was not performed at patient level, so it could not be incorporated into meta-analytic pooling (26).

## Conclusions

This meta-analysis of four randomized controlled trials demonstrates that no statistically significant difference in ischemic clinical outcomes between angiography-derived FFR and pressure-wire-based FFR, while significantly shortening procedural time. Of note, the pooled result for procedural duration was based solely on data from two RCTs. However, this strategy is associated with a higher rate of patient-level and lesion-level PCI. Angiography-derived FFR may serve as an alternative physiological assessment tool in selected clinical settings. Clinicians should be aware of the increased revascularization risk when applying this technique. Further large-scale studies with extended follow-up are required to clarify the long-term consequences of higher PCI rates.

## Data Availability

The original contributions presented in the study are included in the article/Supplementary Material, further inquiries can be directed to the corresponding author.
