# Supplementary material for: Angiography-derived versus wire-based fractional flow reserve for guiding coronary revascularization: a meta-analysis of randomized controlled trials
Source: Front Cardiovasc Med. 2026 Oct 30;13:1969354. doi: 10.3389/fcvm.2026.1969354 (PMC13759418; doi:10.3389/fcvm.2026.1969354)
Supplement: Supplementary file 1 [file Datasheet1.doc]

**Table S1. Search strategy and results in four database.**

| **PubMed** | ("fractional flow reserve"[MeSH Terms] OR "fractional flow reserve"[Title/Abstract] OR FFR[Title/Abstract])  AND  ("quantitative flow ratio"[Title/Abstract] OR QFR[Title/Abstract] OR μQFR[Title/Abstract] OR cFFR[Title/Abstract] OR "angiography‑derived fractional flow reserve"[Title/Abstract] OR "angiography derived FFR"[Title/Abstract] OR vFFR[Title/Abstract] OR FFRangio[Title/Abstract])  AND  (random*[Title/Abstract] OR trial[Title/Abstract]) | 136 |
| --- | --- | --- |
| **Embase** | ("fractional flow reserve":ti,ab OR FFR:ti,ab)  AND  ("quantitative flow ratio":ti,ab OR QFR:ti,ab OR μQFR:ti,ab OR cFFR:ti,ab OR "angiography‑derived fractional flow reserve":ti,ab OR "angiography derived FFR":ti,ab OR vFFR:ti,ab OR FFRangio:ti,ab)  AND  (random*:ti,ab OR trial:ti,ab) | 225 |
| **Cochrane Library** | ("fractional flow reserve":ti,ab OR FFR:ti,ab)  AND  ("quantitative flow ratio":ti,ab OR QFR:ti,ab OR μQFR:ti,ab OR cFFR:ti,ab OR "angiography‑derived fractional flow reserve":ti,ab OR "angiography derived FFR":ti,ab OR vFFR:ti,ab OR FFRangio:ti,ab)  AND  (random*:ti,ab OR trial:ti,ab) | 64 |
| **Web of Science** | TS=("fractional flow reserve" OR FFR)  AND  TS=("quantitative flow ratio" OR QFR OR μQFR OR cFFR OR "angiography‑derived fractional flow reserve" OR "angiography derived FFR" OR vFFR OR FFRangio)  AND  TS=(random* OR trial) | 182 |

**Table S2. Risk‑of‑bias summary and risk‑of‑bias graph for the included randomized controlled trials.**

| **Domain** | FAST‑III | ALL‑RISE | PROVISION | FAVOR III Europe |
| --- | --- | --- | --- | --- |
| **Bias arising from the randomization process** | Low risk | Low risk | Low risk | Low risk |
| **Bias due to deviations from intended interventions** | Some concerns | Some concerns | Some concerns | Some concerns |
| **Bias due to missing outcome data** | Low risk | Low risk | Low risk | Low risk |
| **Bias in measurement of the outcome** | Low risk | Low risk | Low risk | Low risk |
| **Bias in selection of the reported result** | Low risk | Low risk | Low risk | Low risk |
| **Overall risk of bias** | Some concerns | Some concerns | Some concerns | Some concerns |

A	B

C	D

E

Figure S1. Trial sequential analysis plots for secondary clinical outcomes. (A) All‑cause mortality. (B) Cardiac mortality. (C) Recurrent myocardial infarction. (D) Unplanned revascularization. (E) Stent thrombosis. Z‑curve, cumulative Z‑score curve.

A	B

C	D

E	F

G

Figure S2. Funnel plots for the pooled outcomes. (A) MACE. (B) All‑cause mortality. (C) Cardiac mortality. (D) Recurrent myocardial infarction. (E) Unplanned revascularization. (F) Stent thrombosis. (G) Lesion‑level PCI performed. SE, standard error; RR, risk ratio; MD, mean difference.

**Table S3. The result of and certainty of evidence assessment**

| **Certainty assessment** | | | | | | **No of patients** | | **Effect size** | **certainty** |
| --- | --- | --- | --- | --- | --- | --- | --- | --- | --- |
| **No of studies** | **Study design** | **Risk of bias** | **Inconsistency** | **Imprecision** | **Other** | **Pretreatment** | **No pretreatment** | **RR/MD** |  |
| **Primary outcome** | | | | | | | | | |
| 4 | Randomized trials | Not serious | Not serious | Serious | None | 257/3292 | 236/3250 | 1.07(0.91-1.27) | Moderate |
| All-cause mortality | | | | | | | | | |
| 4 | Randomized trials | Not serious | Not serious | Serious | None | 72/3292 | 68/3250 | 1.05(0.75-1.45) | Moderate |
| Cardiovascular mortality | | | | | | | | | |
| 4 | Randomized trials | Not serious | Not serious | Serious | None | 32/3292 | 36/3250 | 0.88(0.55-1.41) | Moderate |
| Repeat myocardial infarction | | | | | | | | | |
| 4 | Randomized trials | Not serious | Not serious | Serious | None | 103/3292 | 98/3250 | 1.04(0.79-1.36) | Moderate |
| Unplanned revascularization | | | | | | | | | |
| 4 | Randomized trials | Not serious | Not serious | Serious | None | 146/3292 | 142/3250 | 1.01(0.81-1.27) | Moderate |
| **Stent thrombosis** | | | | | | | | | |
| 3 | Randomized trials | Not serious | Not serious | Very serious | None | 11/3089 | 12/3052 | 0.91(0.40-2.05) | Low |
| **Lesion‑level PCI performed** | | | | | | | | | |
| **4** | Randomized trials | Serious | Not serious | Not serious | None | 1842/4222 | 1456/4144 | 1.24(1.18-1.31) | Moderate |
| **Patient‑level PCI performed** | | | | | | | | | |
| **3** | Randomized trials | Not serious | Not serious | Not serious | None | 1014/2284 | 811/2258 | 1.24(1.15-1.33) | High |
| **Total procedural time** | | | | | | | | | |
| 2 | Randomized trials | Not serious | Not serious | Not serious | None | 2081 | 2060 | -4.09(-5.76to -2.43) | High |
